# Brain Foods: A Narrative Review of Food Items and Their Impact on Cognition over the Life Course

**DOI:** 10.3390/nu18111779

**Published:** 2026-05-31

**Authors:** Chante Hardaway, Chhavi Tiwari, Atia Bonna, Adegbola Adesogan, Sarah Lindley McKune

**Affiliations:** 1 Department of Community Sustainability, Michigan State University, East Lansing, MI 48824, USA; hardaw16@msu.edu; 2 Department of Environmental and Global Health, University of Florida, Gainesville, FL 32603, USA; chhavitiwari@ufl.edu; 3 Global Food System Institute, University of Florida, Gainesville, FL 32611, USA; atia.bonna@ufl.edu (A.B.); adesogan@ufl.edu (A.A.)

**Keywords:** cognition, animal-sourced foods, plant-based foods, life-course, memory

## Abstract

**Background/Objectives**: Cognitive function is fundamental to daily life, and nutrition is a key modifiable determinant of brain health across the lifespan. While plant-based “brain foods” have been emphasized, the contributions of animal-sourced foods (ASF) to neurodevelopment and cognitive performance remain underexplored. This review synthesizes current evidence on the effects of both plant- and animal-derived foods on cognitive outcomes from early development through older adulthood. **Methods**: A narrative review was conducted focusing on eight major categories of brain-supportive foods—dairy, eggs, seafood, lean meat, berries, leafy green vegetables, nuts, and whole grains. Evidence was evaluated across life stages, considering nutrient bioavailability, dietary patterns, and the interplay between structural, socioeconomic, and environmental factors that influence access to these foods. **Results**: Nutrient-dense foods, including ASF and plant-based sources, support cognitive outcomes across the life course. In early childhood, eggs, meat, and nuts were linked to improved neurodevelopment and reductions in developmental delays, while evidence for seafood and dairy was more mixed. During adolescence and adulthood, berries, walnuts, vegetables, and whole grains were associated with improvements in executive function, verbal reasoning, and mood, with adequate bioavailable protein from ASF remaining important. Among older adults, higher intake of leafy greens, nuts, berries, and moderate seafood consumption correlated with slower cognitive decline and improved memory. Findings were limited by heterogeneous study designs, dietary assessments, and underrepresentation of adolescents and populations in low- and middle-income countries. **Conclusions**: Both animal-sourced and plant-based brain foods uniquely support cognitive development, maintenance, and resilience. While nutritional needs vary across the life course, strong evidence supporting distinct food-based dietary recommendations for cognitive outcomes at different ages, particularly adolescents, remains limited. Current findings suggest stage-specific associations, particularly during early development, but more longitudinal and experimental research is needed. Expanding rigorous, inclusive research will be critical for informing nutrition policies that support lifelong cognitive health.

## 1. Introduction

Cognitive function underpins virtually all domains of daily life, influencing learning, memory, executing functioning, problem-solving, and adaptive behavior [1]. With the global population aging, the prevalence of impairment and neurocognitive disorders has increased substantially, intensifying public health concerns [2,3]. This demographic shift has catalyzed growing interest in modifiable lifestyle determinants of cognitive health. Among these, nutrition has emerged as a central and potentially scalable factor, with converging evidence suggesting that dietary exposures meaningfully influence brain structure [4], function [5], and long-term cognitive trajectories [6]. Beyond its foundational role in somatic health, nutrition directly contributes to neurochemical signaling, synaptic plasticity, neuroinflammation modulation, and oxidative balance processes that collectively shape emotional regulation, mental performance, and resilience to cognitive decline [7].

Nutritional influences on brain health operate across the life course [8]. The human brain undergoes rapid growth and differentiation during prenatal development and early childhood [9], followed by continued synaptic pruning, myelination, and functional specialization through adolescence and adulthood [10]. These dynamic processes are sensitive to nutrient availability and dietary quality. Nutrients such as long-chain omega-3 fatty acids, flavonoids, B vitamins, iron, choline, and high-quality proteins play critical roles in neurogenesis, neurotransmitter synthesis, myelin formation, and protection against oxidative and inflammatory damage [11,12]. Consequently, diets rich in berries, nuts, whole grains, leafy green vegetables, and fatty fish have been frequently characterized as “brain-supportive” due to their bioactive profiles and associations with improved cognitive outcomes [5].

Research on “brain foods” has increasingly emphasized the importance of overall dietary patterns over individual foods alone. Both plant- and animal-sourced foods can contribute essential nutrients relevant to brain development and function. For example, dairy products, eggs, meat, and seafood provide bioavailable forms of vitamin B12, iron, zinc, choline, and complete proteins—nutrients essential for myelination, oxygen transport, methylation processes, and acetylcholine [13]. At the same time, plant-derived foods contribute fiber, antioxidants, polyphenols, and other bioactive compounds that may help reduce oxidative stress and inflammation. Inadequate intake of these nutrients, particularly during sensitive developmental windows, has been associated with impairments in attention, memory, and overall cognitive performance [14]. Thus, a comprehensive evaluation of brain-supportive diets requires consideration of both plant- and animal- derived food sources and the broader dietary context in which they are consumed.

Nutrition also interacts closely with physical growth, particularly during early life, linking cognitive development to stunting and linear growth deficits. Chronic undernutrition and inadequate intake of nutrient-dense foods in the first 1000 days have been associated with delayed brain maturation, impaired synaptic development, and long-term deficits in learning, memory, and attention [15]. Nutrient absorption and bioavailability are critical determinants of these outcomes, as the extent to which nutrients are utilized by the body can vary depending on the food matrix, nutrient composition, preparation methods, and overall diet quality. In some foods, naturally occurring compounds may influence the absorption of minerals such as iron, zinc, and calcium, while other dietary components can enhance nutrient uptake. In adulthood, dietary patterns influence executive functioning, mood regulation, and stress responsivity, potentially through pathways involving neuroinflammation and vascular health. In older adulthood, adherence to nutrient-dense dietary patterns has been associated with reduced risk of cognitive decline and dementia [16,17]. A life-course perspective, therefore, provides a more integrative framework for understanding how specific foods and dietary patterns may exert stage-specific and cumulative effects on cognitive health [18]. However, current evidence does not yet support clearly distinct food-based dietary recommendations for cognitive outcomes across different age groups.

However, access to nutritionally dense, brain-supportive foods is not equitably distributed. Dietary consumption patterns vary widely across regions due to differences in affordability, food system infrastructure, cultural norms, and exposure to environmental or economic shocks [19,20]. These structural determinants shape not only physical health outcomes but also cognitive development, educational attainment, and trajectories of aging across populations [7]. Addressing cognitive health disparities, therefore, requires attention to food system equity alongside individual-level dietary behaviors.

Despite substantial evidence linking nutrition to cognitive function, findings across studies remain heterogeneous. Variability in study design, dietary assessment methodologies, cognitive measurement instruments, and duration of follow-up complicates cross-study comparisons and synthesis [21]. Moreover, much of the literature has historically focused on isolated nutrients rather than whole foods or dietary patterns, limiting ecological validity and translational applicability [22,23]. Greater emphasis on food-based and pattern-based approaches may provide more policy-relevant insights.

This review synthesizes current evidence on eight major categories of brain foods—dairy, eggs, seafood, lean meat, berries, leafy green vegetables, nuts, and whole grains—situated withing within the broader framework of plant and animal-sourced food systems. It summarizes their associations with cognitive outcomes from the first 1000 days of life through older adulthood [24]. By integrating findings across developmental stages and food groups, the review aims to identify areas of convergence, clarify inconsistencies, and highlight priority directions for future research in nutritional neuroscience.

## 2. Materials and Methods

**Figure 1:**
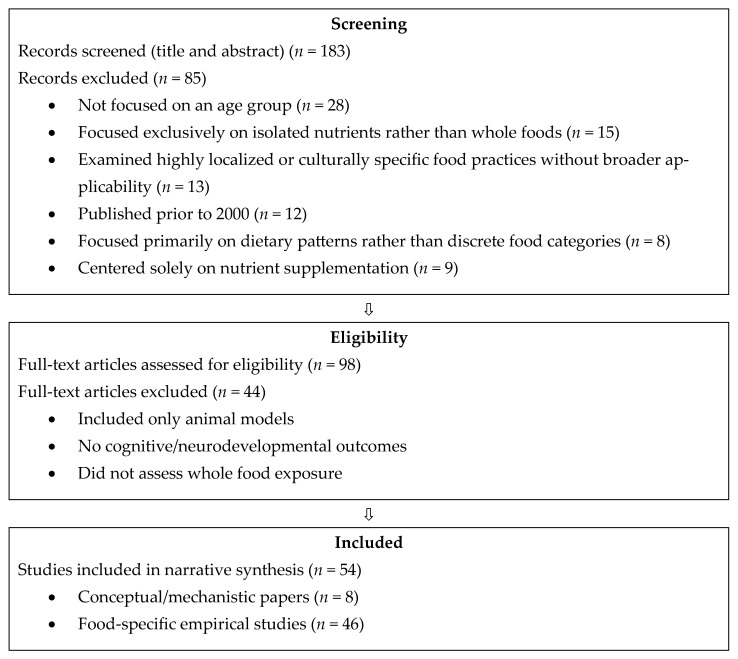
Literature search strategy and study selection process.

This review employed a structured narrative methodology with a multi-stage literature search to identify studies examining associations between commonly consumed whole “brain foods” and cognitive outcomes across the lifespan. The search and screening process is summarized in Figure 1. An initial comprehensive search was conducted in major databases (PubMed, Scopus, and Web of Science) between June and August 2025 to identify relevant literature. Search terms included “brain food”, “brain health”, “cognitive development”, and “lifespan”, yielding 183 articles. We intentionally adopted a focused search strategy to examine evidence on a set of foods repeatedly identified in prior nutritional neuroscience and public health literature as key contributors to brain health across the life course. Rather than conducting a broad scoping review of all dietary factors related to cognition or neurodevelopment, our objective was to synthesize evidence on commonly consumed, nutrient-dense foods that provide nutrients with established relevance to neural structure and function. Titles and abstracts were screened to determine relevance to nutrition and cognitive function across different life stages. Studies were included if they examined whole-food consumption and reported at least one cognitive or neurodevelopmental outcome in infants, children, adolescents, adults, or older adults. Only articles published in English were considered. Duplicate records were identified and removed prior to screening.

A second, more targeted search was conducted to identify studies related to broad food categories based on the food items identified in the first stage search. Search terms included “nuts”, “berries”, “eggs”, “dairy”, “lean meat”, “seafood”, and “cognition”. This allowed for the identification of commonly studied foods within broader dietary categories. The results from the initial search were screened to exclude papers centered primarily on overall dietary patterns rather than specific foods, as well as those focused solely on nutrient supplementation. Based on the findings from this stage, specific food items that appeared frequently and showed potential relevance to cognitive outcomes were identified. A refined search was then conducted using food-specific studies on walnuts, blueberries, and salmon as targeted search terms, alongside cognitive outcomes in adolescents and pregnant women. This step aimed to capture more food-specific evidence and resulted in the inclusion of additional relevant studies.

Following all stages of searching, 54 articles met the eligibility criteria and were included in the review. These comprised 8 broad conceptual papers (e.g., mechanisms linking nutrients to cognition) and 46 food-specific empirical studies. Randomized controlled trials, cohort studies, cross-sectional analyses, and review articles were all considered. Studies that evaluated isolated nutrient supplements, animal-only research, or interventions lacking cognitive outcomes were excluded.

Each included study was reviewed to extract information on sample characteristics, food exposure, cognitive assessments, and major findings. Articles were then grouped by both age categories (first 1000 days, school age, adolescence, adulthood, and older adulthood) and by food category (berries, dairy, eggs, leafy greens, lean meats, nuts, seafood, and whole grains).

Given substantial heterogeneity in study designs, dietary assessment methods, exposure definitions, and cognitive measures, findings were synthesized narratively rather than quantitatively. This approach allowed for the integration of mechanistic evidence, observational findings, and intervention studies to identify consistent themes and gaps in the literature.

## 3. Results

Brain foods are dietary components that provide nutrients known to influence neuronal structure, synaptic signaling, and long-term cognitive function. Evidence from nutritional neuroscience demonstrates that specific nutrients modulate molecular pathways involved in synaptic plasticity, oxidative balance, mitochondrial performance, and neuroinflammation, thereby shaping cognitive outcomes across the lifespan [7]. Based on the mechanisms identified and evidence found, to present results, we have categorized brain foods into two broad categories, ASF and plant-based foods, each contributing distinct biochemical substrates essential for optimal brain function [7,25]. Based on our literature review, the effects of brain foods vary depending on life stage, reflecting differences in neurodevelopment, metabolic needs, and vulnerability to oxidative or inflammatory stressors. Therefore, the following sections also summarize findings from infancy through older adulthood for each food category. A detailed description of the studies included by food items and age groups is presented in Table 1.

### 3.1. Animal-Sourced Foods

#### 3.1.1. Eggs

In addition to protein and essential vitamins (including B6, B12, and folate), eggs supply choline, phospholipids, and omega-3 fatty acids, which support acetylcholine synthesis, membrane fluidity, and neuronal signaling [12]. Nutrients that enhance synaptic plasticity, particularly through modulation of brain-derived neurotrophic factor (BDNF) and membrane structure, are central to learning and memory processes [7,26]. They also provide lutein and zeaxanthin, carotenoids that accumulate in brain tissue and are associated with neural efficiency and protection against oxidative stress [27].

In a nutrition trial in Ecuador by [28], a 1 egg per day intervention among children aged 6–9 months was associated with increased plasma concentration of key nutrients involved in neurodevelopment, including choline and its biomarkers, betaine, methionine, and docosahexaenoic acid (DHA). A behavior change cluster randomized controlled trial that increased egg consumption among children in Burkina Faso [29] found that children who consumed eggs regularly had reduced odds of delays in communication, motor development, and social functioning [30]. An ongoing randomized trial is evaluating the impact of daily egg consumption from 9 to 18 months on growth and cognitive outcomes [26]. The other age group where the effect of egg consumption on cognitive outcomes has been noted is older adults and the elderly population [31]. Evidence from reviews and observational studies suggests that moderate egg consumption is associated with slower rates of cognitive decline compared with low intake [32]. One randomized controlled trial reported improvements in reaction time following egg consumption, potentially linked to enhanced cholinergic signaling [31,32]. However, for school-aged children and adolescents, evidence remains limited. We did not find any review directly examining egg intake and cognitive outcomes in school-age children or adolescents, or older age groups.

#### 3.1.2. Dairy

In addition to high-quality proteins, dairy provides a range of vitamins and minerals, including calcium, phosphorous, and magnesium, that support mitochondrial efficiency and reduction of oxidative stress [33]. They are also rich in B vitamins (particularly B2 and B12), which are essential for energy metabolism and neural function, as well as vitamin D (in fortified products), potassium, and bioactive peptides that contribute to cellular signaling and antioxidant defense. Oxidative stress is known as a major contributor to impaired synaptic function, which positions dairy-derived antioxidants and micronutrients as potentially neuroprotective [34]. The cognitive effects of dairy may relate to their supply of B vitamins, calcium, vitamin D, and milk polar lipids, which support neuronal membrane integrity and neurotransmitter synthesis [35]. However, high saturated fat content in some dairy products may counteract these benefits via vascular or inflammatory pathways [36]. Evidence linking dairy consumption to cognitive outcomes was inconsistent across age groups. Maternal dairy intake has been associated with improved offspring neurodevelopment, likely due to high-quality protein, calcium, and micronutrients that reduce oxidative stress and support cellular growth [37].

Evidence from systematic reviews, observational studies, and supplementation trials suggests mixed effects of dairy intake during early life. Reviews indicate that excessive dairy or high-protein intake in infancy may negatively affect aspects of neurodevelopment or infant behavior, while modest dairy-based supplementation with adequate micronutrient content may result in small, short-term improvements in motor development and temperament [36,37,38]. A 2-arm cluster randomized controlled trial in Yemen [39] found that providing children aged 6–18 years with milk as a supplement to high-energy biscuits led to improvements in cognition, literacy, and numeracy, alongside reductions in conduct problems. In a large Japanese cohort, yogurt consumption during infancy was associated with a reduced risk of developmental delays across several developmental domains, though benefits were not observed at higher intake frequencies [40]. In adults and older adults, observational and randomized trials showed either no association or mixed effects, with yogurt consumption being the only dairy product consistently linked to higher cognitive scores in one large observational study [41,42].

#### 3.1.3. Meats

Meats provide bioavailable iron, B vitamins (particularly B6 and B12), as well as essential amino acids required for neurotransmitter synthesis and energy metabolism [43]. They also supply lipids and cholesterol that support myelin formation, facilitating efficient nerve signal transmission and neuronal protection. Adequate mitochondrial function is emphasized as a prerequisite for sustained cognitive activity, linking these nutrients to improved neuronal resilience [7,44]. It is important to distinguish between different types of meat, as their nutritional profiles and health implications differ. Lean meats, such as poultry (e.g., chicken and turkey), are generally lower in saturated fat, whereas red meats (e.g., beef and lamb) contain higher levels of saturated fats and have been associated with different cardiometabolic risks [45,46,47]. Processed meats further differ due to added preservatives, sodium, and altered fat composition, which may influence health outcomes differently from unprocessed meat sources [48,49,50].

Intervention studies comparing different types of meat (including lean meats such as poultry and red meats such as beef and pork) generally showed similar cognitive improvements across groups, suggesting that protein adequacy from meat rather than meat type may be the key factor [51]. Only a few studies have examined meat consumption in early life with explicit cognitive endpoints, despite its role as a nutrient-dense food. In a randomized feeding trial in Kenya, 555 primary school children were assigned to one of four interventions—meat, milk, or energy (oil) supplements to a plant-based diet called githeri that included beans and corn for 21 months [52]. Children in the meat supplementation group showed significantly greater gains in non-verbal reasoning than all other groups. Both the meat and energy groups outperformed the control and milk group on arithmetic ability and Raven’s Progressive Matrices, which reflects greater fluid intelligence, abstract reasoning, and the ability to solve novel problems. A randomized trial in undergraduate women found improved attention and planning in groups receiving either lean beef or non-beef protein sources, suggesting that high-quality protein and micronutrients may support executive function [51]. In older adults, results are mixed. In the MedPork trial, weekly consumption of lean pork improved processing speed in older adults relative to a low-fat control diet, while memory and attention remained unchanged [53].

The foregoing suggests that a growing body of evidence indicates that, like dairy and eggs, moderate meat consumption by infants contributes to improved cognition, but the literature on effects at older ages is less consistent. Most studies included in this review did not clearly differentiate between meat categories, limiting interpretation of whether observed cognitive effects are attributable to specific meat types or overall protein intake.

#### 3.1.4. Seafood

Seafood is a primary source of long-chain omega-3 fatty acids, particularly Docosahexaenoic Acid (DHA). It also provides high-quality protein, iodine, selenium, zinc, vitamin D, and B vitamins (including B12), all of which contribute to neural development, neurotransmitter synthesis, antioxidant defense, and thyroid function. DHA plays a key role in enhancing synaptic plasticity, regulating inflammatory pathways, and supporting membrane architecture. Omega-3 fatty acids are among the most important nutrients for cognitive enhancement due to their effects on BDNF expression and neuroinflammatory modulation [7,54].

Evidence for seafood consumption and cognition in early life was weak and inconsistent. Randomized trials showed minimal effects on mental health outcomes, though small improvements in reaction time were noted among infants [55]. A recent review concluded that evidence supporting seafood-related cognitive benefits across childhood and adolescence remains very low [56]. A large meta-analysis reported that fish consumption up to 150 g/day was associated with reduced risk of dementia and cognitive impairment. For Alzheimer’s disease, protective effects plateaued at lower intake levels, suggesting a nonlinear relationship between intake and risk [57]. The lack of consistent findings may reflect variability in dosage, exposure timing, and background diet.

Dietary fat composition is an important factor in understanding the relationship between diet and cognitive health [58]. Unsaturated fats, particularly omega-3 fatty acids from seafood and plant sources, are associated with improved synaptic plasticity and reduced neuroinflammation [59]. In contrast, high intake of saturated fats, commonly found in red and processed meats, may negatively impact cognitive function through vascular and inflammatory pathways [60,61]. These differences highlight the importance of considering fat quality alongside food sources when evaluating dietary effects on cognition.

### 3.2. Plant-Based Foods

#### 3.2.1. Berries

Berries contain flavonoids and polyphenols that mitigate oxidative stress and modulate signaling cascades associated with synaptic plasticity. Antioxidants can counteract oxidative injury, a major driver of cognitive impairment, and thereby support learning and memory [7,62].

Across the lifespan, consumption of berries was found to show selective cognitive benefits, with effects varying by age, dose, and cognitive domain. In children and adolescents, acute or short-term supplementation with wild blueberries or berry-derived beverages was associated with improvements in executive function and memory [63,64] and mood [65], while null effects were reported for fatigue, sleep, and broader mood outcomes in athletic adolescents. Most studies begin in mid-childhood (around 7–10 years), and data for younger ages remains limited. Among adults, multiple randomized controlled trials reported modest improvements and outcome-specific cognitive effects. Blueberry and grape juice supplementation has been associated with improved mood and immediate spatial memory, though findings are inconsistent across studies and cognitive domains [65,66]. Among older adults, berry consumption is consistently associated with slower cognitive decline, improved working memory, and enhanced neural activation on imaging studies [67]. Grape extract and cherry juice interventions were found to be associated with improvements in attention, language, executive function, and both immediate and delayed memory [68,69]. Some evidence suggests potential protection against dementia when intake is established earlier in adulthood, although long-term trials are still emerging [70].

#### 3.2.2. Whole Grains

Whole grains provide complex carbohydrates, B vitamins, and dietary fiber that support metabolic stability and gut/brain hormonal communication. Hormones such as ghrelin, leptin, and GLP-1, described as regulators of synaptic activity and memory processes, are influenced by dietary patterns rich in whole grains [71]. Whole grains provide steady glucose, B vitamins, and fiber that support energy metabolism and gut/brain signaling [72].

Higher whole grain intake has been associated with reduced depression and anxiety, with some evidence for improved executive function and memory [72,73]. In a Chicago-based cohort, higher whole grain consumption among African American adults was linked to slower global cognitive decline, particularly in memory and processing speed [74].

#### 3.2.3. Leafy Green Vegetables

Leafy greens supply folate, carotenoids, vitamin K, and other antioxidants that contribute to neuronal maintenance and reduce oxidative load. Antioxidants preserve synaptic integrity and modulate inflammatory pathways implicated in cognitive decline [75].

In Japanese middle and high school students, regular consumption of green and yellow vegetables was associated with fewer depressive symptoms [76], suggesting potential benefits for mental health during this vulnerable developmental period. In a longitudinal study of older adults, consumption of one to two servings of leafy greens per day was associated with cognitive performance equivalent to being about 11 years younger [75].

#### 3.2.4. Nuts and Seeds

Nuts and seeds supply unsaturated fatty acids, vitamin E, and polyphenols that stabilize neuronal membranes, protect against oxidative damage, and support mitochondrial performance. These biological effects enhance synaptic efficacy and reduce metabolic stress in neural tissue [77,78].

Nut consumption was strongly associated with enhanced cognitive development when consumed during pregnancy and with better memory and executive outcomes later in life [79]. Intervention trials in young adults showed improvements in verbal reasoning following walnut supplementation [80]. Walnut consumption has been linked to improved reaction time and attention in school-aged children in Korea, although no associations were observed for verbal or visual memory [81]. A growing literature demonstrates that higher nut intake is associated with reduced risk of cognitive impairment, improved delayed memory, and enhanced reasoning [82,83]. Most studies are observational, but findings are generally consistent.

### 3.3. Regional Variation

The current evidence linking brain foods to cognitive outcomes is geographically skewed. As summarized in Table 1, the majority of studies have been conducted in high-income countries (HICs), particularly the United Kingdom, United States, Australia, Spain, Italy, Japan.

Relatively few randomized or longitudinal studies have been implemented in low- and middle-income countries (LMICs), and those that exist (egg interventions in Burkina Faso and India, dairy supplementation trials in India, and the meat supplementation study in Kenya) are limited in number and often smaller in scale. As a result, the populations most affected by limited access to brain-relevant nutrients are underrepresented in the clinical evidence base used to inform dietary recommendations. This creates a paradox: regions with the lowest per capita intake of nutrient-dense foods and the highest exposure to developmental risk factors have the least context-specific evidence guiding nutrition policy for cognitive health.

**Figure 2:**
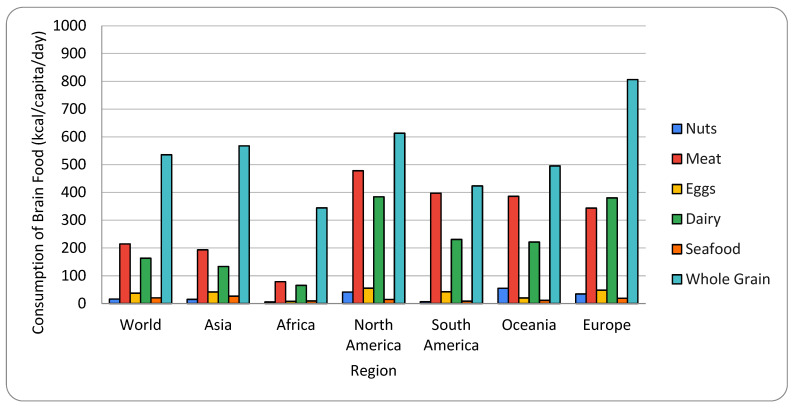
Consumption of brain foods in kcal/capita/day by global region (Datasource: FAOSTAT, 2023 [20]). Note that consumption data for Berries and Leafy vegetables were not available on FAOSTAT.

Understanding the regional focus of these studies can also be explained by consumption patterns of these foods across regions and is essential for contextualizing nutritional disparities and their potential implications for cognitive health. Analyzing data from the FAO Food Balance Sheets (FAOSTAT, 2023 [20]), clear regional differences emerge across major food groups, particularly for ASF, nuts, and seafood (Figure 2). These differences reflect long-standing inequalities in food access shaped by socioeconomic conditions, cultural norms, and environmental constraints.

Across most regions, intake of dairy, eggs, and lean meats remains substantially lower in South Asia and sub-Saharan Africa compared to North America and Europe. These gaps are especially relevant given the role of ASF in supplying bioavailable iron, choline, vitamin B12, and high-quality protein nutrients, with direct implications for brain development, synapse formation, and neurotransmitter synthesis. Similarly, seafood consumption is markedly uneven, with Asia consuming significantly higher quantities relative to Africa, Latin America, and parts of Europe, despite strong evidence linking omega-3 fatty acids to cognitive outcomes.

Plant-based brain foods show parallel disparities. Nuts and seeds, rich in polyunsaturated fats and antioxidants, are consumed at relatively low levels in South America and Africa. Reliable global intake data for berries and leafy green vegetables are limited, but regional dietary surveys suggest large differences in consumption frequency and availability [20].

These patterns highlight a persistent nutritional divide. Regions experiencing higher food insecurity are often the same regions facing poorer educational outcomes, higher exposure to psychosocial stressors, and increased vulnerability to climate shocks, and are disproportionately limited in access to nutrient-dense foods essential for optimal cognitive development. This may contribute to long-term disparities in learning, mental health, and cognitive aging. Table 1 summarizes the available consumption data used in this review and illustrates the magnitude of these differences across geographic regions.

**Table 1:** Studies included and a summary across food groups by age.

Food	Age Group	Types of Intervention	Country	Findings
Berries	First 1000 days			
	School-age children (7–10 years)	300 g freeze blueberry powder [64] Blueberry drink [64,65]	UK (3)	Improved executive function during demanding elements of tasks [63]. Improvement in delayed recall of a previously learned list of words [64]. Increased positive effect on mood [65].
	Adolescents (16–19 years)	60 mL cherry concentrate drink [84]	UK (1)	No change in fatigue, sleep, or mood [84].
	Adults (18–50 years)	Blueberry drink 335 mL concord grape juice daily for 6 to 12 weeks	UK (2)	Increased positive effect on mood [65]. Improved immediate spatial memory. No difference in mood [66].
	Older adults (55–75 years)	250 mg/d of Cognigrape-V extract powder for 12 weeks [68]	Italy (1)	Improved attention, language, and immediate and delayed memory.
	Older adults (≥70 years)	200 mL/d cherry juice for 12 weeks [69]	Australia (1)	Improved cognition in memory and executive function. No effect on vitamin C and inflammatory markers.
Dairy	First 1000 days	Review of articles [37] Review of 7 interventions [36] Population-based observational study [40] Daily supplementation, for 180 d, with milk–cereal mix that provided about 125 kcal of energy, 30–45% energy from fats, and 80–100% RDA of growth-relevant multiple micronutrients [38]	India (1) Japan (1) UK (1)	Reduced cognitive development of the offspring [37]. Polar lipids in milk do not have solid research on how it impacts the cognitive development of infants, but it does help with the cognitive health of mothers consuming more dairy products with these polar lipids [36]. Reduced their risk of developmental delay in all of the ASQ3 domains, and when over or equal to 5 times a week, the reduced risk was no longer present for fine motor skills or personal-social skills [40]. Supplementing with a modest amount of protein and multiple micronutrients may lead to short-term small improvements in motor function and infant temperament. There appears to be no advantage of supplementing with high protein; rather, negative effects on infant behavior were observed [38].
	School-age children (6–18 years)	2-arm longitudinal c-RCT where children aged 6–18 yrs received high-energy biscuits (HEB) or milk [39] Children received one of four feeding interventions: Meat, Milk, Energy, or Control [52]	Yemen (1) Kenya (1)	Children’s cognition, literacy, and numeracy scores improved. The intervention also reduced conducts problem in children, severe anxiety in caregivers, and household severe food insecurity [39].
	Adults	Review of 6 articles [85]		Found either no correlation between dairy and cognitive decline or that there was an increased risk of cognitive decline with increased consumption [85].
	Older adults (55–87 years)	Population-based, observational survey [41] Randomized parallel RCT 148 g/day, 282 g/day, and 546 g/day of dairy consumption for 2 years [42] Meta-analysis of 38 studies [86] Meta-analysis and systematic review of 8 studies [87]	US (1) Spain (1)	Cognitive scores were higher (40.03 ± 0.64 vs. 36.28 ± 1.26, *p* = 0.017) in participants consuming dairy yogurt than not [41]. No clear association between the intake of commonly consumed dairy products and cognitive performance in older adults [42]. Higher total dairy was associated with a reduced risk of cognitive decline and improved global cognitive function [86].
Eggs	First 1000 days	1 egg per day from 9 to 18 months [26] 1 egg per day for 6 months in children 6–9 months [28] Intervention to consume eggs daily, some given full, partial, or some intervention [30]	India (1) Burkina Faso (1) Ecuador (1)	Currently being completed [26]. Consistent egg consumption found to lower the odds of falling below the cut-off in communication skills, gross motor, and personal social skills. Increased egg consumption improved problem-solving [30]. Egg intervention increased plasma concentrations, with increased choline, betaine, methionine, and docosahexaenoic acid.
	Adults	Review of 12 Studies [88] Low got half an egg per week, intermediate got 1.5 eggs per week, and high got 2+ eggs per week [32]	US (1)	Five showed that egg consumption slowed rates of cognitive decline, but four showed there was no correlation, and two showed no connection to memory. The RCT showed improved reaction time with egg consumption [88]. Intermediate egg consumption showed significantly slower rates of cognitive decline than the low egg group [32].
Green Vegetables	Adolescents (13–18 years)	Observational perspective surveying breakfast patterns [76]	Japan (1)	Regular consumption of green and yellow vegetables has significantly lower depressive symptoms [76].
	Adults (18–30 years)	Observational longitudinal study [89]	China (1)	Total vegetable consumption is associated with better cognitive performance. Looking at subgroups, tomatoes, dark green and deep yellow vegetables, and avocado were associated with better cognitive performance [89].
	Older adults (58–99 years)	No intervention, just observing and encouraging vegetable consumption [75]	US (1)	Consumption of green leafy vegetables was positively and significantly associated with a slower rate of cognitive decline. In those who consumed 1–2 servings per day, it was the same as being 11 years younger mentally [75].
Meats	School-age children	Children received one of four feeding interventions: Meat, Milk, Energy, or Control [52]	Kenya (1)	Children supplemented with meat outperformed children in the control group on arithmetic ability.
	Adults (20–21 years)	Participants were randomized to a meal of beef vs. non-beef protein at 100 g protein/meal, 3×/week for 16 weeks [51]	US (1)	Both groups had improvement in spatial working memory strategy, attention, and planning speed [51].
	Older adults (45–80 years)	MedPork diet 250 g lean pork/week vs. low-fat diet for 8 weeks, then 8-week washout [53]	Australia (1)	MedPork has a greater increase in processing speed performance but not memory, attention, or planning [53].
	Older adults (≥65 years)	Randomized to a meal of pork or chicken 4×/week for 12 weeks [90] Lean red meat 160 g/serving vs. carbohydrate 3×/week for 24 weeks [91]	Australia (2)	The control group (chicken) has greater improvements in verbal learning and memory scores at week 6 but not 12 [90]. There were no differences for global cognition or executive function; working memory and learning were better under the control [91].
Nuts	First 1000 days	Children followed from birth until 8 years old, dietary intake questions asked 2× during pregnancy and at 1.5, 5, and 8 years old [79]	Spain (1)	Higher maternal consumption of nuts in early pregnancy was associated with enhanced cognitive development at 1.5, 5, and 8 years old [79].
	School-age children (6–18 years)	Dietary intake questions in a semi-quantitative food frequency questionnaire [81]	Korea (1)	Improved cognitive region time consistency and attention function, but no association between verbal and visual memory tests [81].
	Adolescents (18–25 years)	56% white, three slices of banana bread with walnuts (60 g of ground walnuts/day) consumed for 8 weeks, then 6 weeks of washout, then 8 weeks placebo (and vice versa) [80]	US (1)	Effect observed in verbal reasoning, but nothing for memory, nonverbal reasoning, or mood [80].
	Older adults (50+ years)	Observational study of nut consumption [92] Review of 15 papers [82]	China (1)	Higher nut consumption is associated with delayed memory loss [92]. A total of 13 out of the 15 studies showed a positive association between nut consumption and cognitive performance, but not always in all cognitive assessments [82].
	Older adults (60+ years)	Longitudinal study observing nut consumption [83] Observational study of nut intake [93]	China (1) US (1)	Less cognitive impairment for the highest nut intake group of ≥70 g per week [83]. Any walnut consumption has greater scores at baseline, with no association with cognitive changes [93].
Seafood	First 1000 days	Randomized jar food given containing either rapeseed oil, oily fish, or corn oil for 5 months [55]	Germany	No statistical difference in mental development, but reaction time was statistically different, being 4–6 ms faster than the corn oil control [55].
	School-age children	Review of 14 papers [56]		Across 14 papers looking at cognitive development, they found very low evidence to suggest that seafood consumption improved cognitive development, but found nothing for behavior, language, movement/physical development, mood, adhd, or autism [56].
	Adolescents	Review of 14 papers [56]		Across 14 papers looking at cognitive development, they found very low evidence to suggest that seafood consumption improved cognitive development, but found nothing for behavior, language, movement/physical development, mood, adhd, or autism [56].
Whole Grains	Adults	Review of 3 of papers [73]		Looking at 3 studies, an intake of 30–45 g of whole grains/day was associated with better cognitive function.
	Adults	Review of 23 studies [72]		Out of the 23 studies reviewed (4 RCT and 19 Observational), there was no conclusive evidence that whole grains reduced cognitive decline, but there was a consistent association with higher levels of whole grain intake and reduced scores for mood disorders, including depression and anxiety [72].
	Older adults (65+ years)	Longitudinal observation study of eating habits and recorded daily consumption [74]	US	In African American participants, higher consumption of whole grains was associated with a slower rate of global cognitive decline; the highest quintile of whole grain consumption had a slower rate of decline in memory speed and cognition [74].

## 4. Discussion

This review synthesized evidence on eight foods commonly identified as supportive of brain health across the life course: dairy, eggs, seafood, meat, berries, leafy green vegetables, nuts, and whole grains, selected based on their frequent identification in prior literature as nutrient-dense contributions to brain health. These foods provide nutrients central to neuronal structure and function—including choline, iron, vitamin B12, long-chain omega-3 fatty acids, B vitamins, carotenoids, and polyphenols—which influence synaptic plasticity, neurotransmission, mitochondrial efficiency, and oxidative balance [7,25]. Some dietary patterns, for example, the Mediterranean diet, align well with these principles. The Mediterranean diet emphasizes high intake of fruits, vegetables (particularly leafy greens), whole grains, nuts, and olive oil; moderate consumption of fish, dairy, and eggs; and limited intake of red and processed meats [94]. Evidence suggests that cognitive benefits are derived from this balanced dietary approach rather than reliance on any single food group [74,95]. Building on these principles, the MIND diet (Mediterranean-DASH intervention for neurodegenerative delay), developed by researchers at Rush University and Harvard University, was specifically designed as a brain-healthy dietary pattern aimed at reducing cognitive decline and lowering the risk of Alzheimer’s disease. Although the biological plausibility is strong, observed cognitive effects vary by life stage and study design.

Dietary fat composition is also an important factor in cognitive health [58]. Unsaturated fats, particularly omega-3 fatty acids from fish and plant sources, are associated with improved neuronal function and reduced neuroinflammation [96,97].

During the first 1000 days and early childhood, nutrient-dense foods appear particularly relevant to neurodevelopment. Intervention studies provide compelling evidence. For example, egg supplementation in Burkina Faso reduced developmental delays in young children [29,30], maternal nut intake has been associated with improved cognitive outcomes in offspring [79], and the meat supplementation in Kenyan school children demonstrated significant gains in arithmetic and non-verbal reasoning [52]. Dairy supplementation trials have shown modest benefits when micronutrient adequacy is achieved, though excessive protein intake may confer no additional advantage [36,38]. Evidence linking seafood intake to early cognitive development remains inconsistent, with reviews concluding that certainty is low [56]. The literature suggests that meat, dairy, and egg consumption by infants contributes to improved cognition, though the evidence for seafood consumption is mixed. This may be because ASF supplies infant cognition-dependent micronutrients (iron, zinc, iodine), and B vitamins (B12, B6, folate, choline, and riboflavin) that are either in more bioavailable forms or are lacking from plants (B12). Further, ASF, like meat, increase bioavailability of plant micronutrients like iron and zinc.

An additional explanation for the improvement in infant cognition by ASF may relate to differences in protein quality between plant and animal foods. Animal foods contain the full complement of amino acids, but plant foods do not. Insufficient provision of any single amino acid from the maternal diet can hinder protein synthesis by the fetus and can have deleterious effects on fetal brain development [98]. Consequently, in the developing fetus, which only receives amino acids, not protein, through the placenta [98], a maternal diet deficient in ASF could hinder protein synthesis by the fetus and could have deleterious effects on fetal brain development similar to those induced by the omission of proteins [98,99].

In adolescence and adulthood—life stages characterized by continued frontal lobe maturation and increasing cognitive demands—evidence is more limited and heterogeneous. Berry and walnut interventions have demonstrated domain-specific improvements in executive function or verbal reasoning [63,80], while vegetable and whole grain intake have been associated with better cognitive performance and mood outcomes in observational studies [72,89]. Trials comparing meats with other protein sources suggest that adequate total protein intake is critical for cognitive outcomes [51], but the sources of protein matter, as meat provides more bioavailable amino acids, iron, zinc, vitamin B12, and other micronutrients essential for neurodevelopment. Plant-based foods contribute essential nutrients, fiber, and bioactive compounds that support gut microbiota, reduce inflammation, and play a critical role in long-term cognitive and overall health [100].

Among older adults, higher intake of leafy green vegetables and nuts has been associated with slower cognitive decline [75], and berry interventions have demonstrated improvements in memory and executive function. Moderate seafood consumption has been associated with a reduced risk of cognitive impairment and dementia [57]. Dairy findings remain mixed, with yogurt showing the most consistent positive associations in observational data [41] while randomized trials often report null effects [42].

Several limitations exist in interpretation. Heterogeneity in dietary assessment, cognitive testing, exposure duration, and baseline nutritional status contributes to inconsistent findings. Most evidence derives from observational studies, limiting causal inference. Furthermore, adolescence is notably underrepresented [7]. An additional limitation of the current literature is the lack of differentiation between whole foods and ultra-processed foods. Processed foods, including processed meats and refined products, are increasingly associated with adverse cognitive and mental health outcomes due to high levels of sodium, additives, and unhealthy fats [49,50,101]. Future research should more clearly distinguish between minimally processed and highly processed dietary patterns to better understand their independent effects on cognitive health.

A critical and often overlooked issue is the geographic concentration of research in high-income countries. Most of the intervention and longitudinal studies were conducted in North America, Europe, Australia, and parts of East Asia, whereas populations in low- and middle-income countries—where access to livestock-derived ASF (meat, eggs, and dairy), nuts, and seafood is often limited—remain underrepresented. This imbalance is particularly concerning given global disparities in nutrient-dense food access and the potential implications for cognitive development and aging trajectories.

Future research should prioritize longitudinal and randomized designs across diverse populations, incorporate objective biomarkers of nutrient status, and evaluate foods within broader dietary patterns. Expanding research in underrepresented life stages and regions will be essential to ensure that dietary guidance for cognitive health is both evidence-based and globally relevant.

## 5. Conclusions

Brain foods provide essential nutrients that influence cognitive outcomes from early development through older adulthood. The evidence reviewed suggests that both animal-sourced and plant-based foods can play a role in neurodevelopment, cognitive function, and the maintenance of cognitive health, with animal-sourced foods being important supplements for infant neurocognitive development. However, these associations are not uniform, with some food groups showing mixed or limited evidence. Important gaps remain, including a relative lack of studies in adolescents, variability in study designs and outcome measures, and limited representation of populations from low- and middle-income countries. These factors constrain the generalizability of current findings. Future research using more standardized and longitudinal approaches, as well as more diverse populations, is needed to better understand these relationships.

## Data Availability

No new data were created or analyzed in this study. Data sharing is not applicable to this article.

## Abbreviations

The following abbreviations are used in this manuscript:

ASF	Animal-sourced foods
BDNF	Brain-derived neurotrophic factor
DHA	Docosahexaenoic Acid
FAO	Food and Agriculture Organization
GLP-1	Glucagon-like peptide-1
HIC	High-income countries
LMICs	Low- and middle-income countries
RCT	Randomized-controlled trial
UK	United Kingdom
US	United States
